# A new lens on wellbeing: psychometric evaluation of the Wellbeing scale in India

**DOI:** 10.3389/fpsyg.2026.1898104

**Published:** 2026-10-07

**Authors:** Kamlesh Singh, Naina Nagpal, C.K. Jaseel

**Affiliations:** Department of Humanities and Social Sciences, Indian Institute of Technology Delhi, New Delhi, India

**Keywords:** eudaimonia, hedonia, physical wellbeing, social wellbeing, subjective wellbeing

## Abstract

**Introduction:**

Wellbeing is widely viewed as a multi-dimensional construct, encompassing factors like hedonia, eudaimonia, financial contentment, physical health, and social connectedness. The Wellbeing Scale integrates these dimensions into a unified framework for measuring wellbeing. While wellbeing research is growing in India, there is limited validation of the scales used. This study aimed to validate the English and Hindi versions of the Wellbeing Scale in the Indian context.

**Methods:**

A convenience sample of 1795 adults (M age = 26.08 years) participated, with 986 completing the survey in English and 809 in Hindi. CFA was conducted for both language versions, and measurement invariance across language and socio-demographic variables was tested. Additionally, MANOVA examined the influence of socio-demographic factors on wellbeing domains.

**Results:**

CFA results indicated that the bifactor model confirmed the best fit for both language versions, while the higher-order factor structure demonstrated better measurement quality, reliability, and validity. Positive correlations were found between all factors and life satisfaction and positive feelings. Hedonic wellbeing was negatively associated with negative experiences. Measurement invariance testing supported the higher-order factor structure across languages and demographic variables. Wellbeing scores were significantly influenced by education, family income, and residence type.

**Discussion:**

Both the Hindi and English versions of the Wellbeing Scale are validated for use in the Indian context and are suitable for comparing wellbeing across demographic groups.

## 1 Introduction

The relevance of the concept “wellbeing” is distinctly evidenced in the argument presented by McCallum and Price (2016) indicating that “wellbeing” has become “something everyone seemingly aims for, and arguably has a right to” (McCallum and Price, 2016, p.2). Nevertheless, despite extensive research and scholarly endeavors in the realm of psychology spanning numerous decades, a unanimous definition of wellbeing remains elusive. Drawing an apt analogy, Carter and Andersen (2023), in their textbook on wellbeing in educational context, likened the notion of wellbeing to a chameleon that alters its form. Despite the growing acknowledgment of the significance of wellbeing, it remains regrettable that a consensus concerning its quantification continues to elude the scholarly community (Clark, 2016; Jayawickreme et al., 2012). Nonetheless, this absence of accord has spurred the development of an assortment of conceptual frameworks and theories addressing wellbeing, along with corresponding measurement tools. A recent comprehensive analysis of scales gauging subjective wellbeing (SWB) conducted by Ong et al. (2021) identified a total of 56 measures derived from peer-reviewed articles published between 2015 and 2019 in MEDLINE, EMBASE, and PsychInfo. It seems fair to conclude that a wealth of tools for assessing subjective wellbeing is available. However, the diverse perspectives on the conceptual underpinnings of this construct have resulted in a fragmentation of current research on subjective wellbeing, rendering it disjointed and non-convergent (Lui and Fernando, 2018).

Lui and Fernando (2018) in their study identified certain factors impeding advancements in the realm of research concerning subjective wellbeing. Drawing upon an exhaustive examination of existing literature, they asserted that the present conceptualization and evaluation of subjective wellbeing tend to center around discrete constituents of Subjective Wellbeing. Despite previous research showcasing the significance of encompassing hedonia - the experience of pleasure and positive affect (Ryan and Deci, 2001), eudaimonia - living in accordance with one's values and realizing personal potential (Waterman, 1993), and psychological wellbeing - a broader construct including emotional, social, and functional aspects of mental health (Ryff, 1989) as dimensions of SWB, and independently highlighting the essential roles played by financial contentment and physical health in establishing an empirical comprehension of SWB, they found that there is a lack of measurement instruments had integrated these elements cohesively within a unified theoretical framework. An additional observation they made pertains to the tendency of subjective wellbeing measures to disproportionately overlook the aspect of satisfaction derived from social connectedness, a facet of wellbeing outlined by Keyes (1998), the omission of which they posited might be attributed to an overreliance on an individualistic cultural paradigm, thereby attenuating emphasis on domains of wellbeing that hold distinct relevance within collectivistic cultures (Cooke et al., 2016). Furthermore, they pointed out the ongoing debate surrounding the underlying factor structure of SWB, evidenced by conflicting findings across studies (e.g., Ryff, 1989; Vella-Brodrick and Allen, 1995; Jovanović, 2015b; Longo et al., 2016).

Lui and Fernando (2018) embarked on the development of an enhanced multidimensional measurement tool for subjective wellbeing (SWB) to address these existing gaps. This novel instrument named the “Wellbeing scale” (WeBS) aimed to evaluate the overarching state of wellbeing by leveraging items sharing a common underlying variance, while concurrently delineating distinct domains within this construct, encompassing financial, physical, social, hedonic, and eudaimonic facets. In their conceptualization, subjective wellbeing is defined as “a multidimensional construct encompassing individuals' emotional, psychological, and social evaluations of their lives” (Lui and Fernando, 2018, p. 136). The formulation and validation of WeBS for wellbeing drew guidance from both the Hierarchy of Needs theory and the robust empirical foundation concerning diverse dimensions of wellbeing. This newly devised WeBS comprises of 29 items. The analysis unveiled that the WeBS could be understood as comprising a global factor for SWB shared across domains, accompanied by five domain-specific factors. The results of the study also demonstrated a significant positive correlation between the overall WeBS scores and the scores of life satisfaction. Conversely, these scores exhibited significant negative correlations with symptoms of depression and anxiety.

In their study, Lui and Fernando (2018) also points out the significance of ethnicity within the landscape of wellbeing research (Rao and Donaldson, 2015) and incorporated participants from diverse ethno-cultural backgrounds, including Asians, into their investigation. However, it is crucial to note that irrespective of the racial or ethnic categorization of participants, all individuals under study were residents of America. To assert the generalizability and validity of the scale in a dissimilar socio-cultural milieu solely based on this premise would be an oversimplification. Particularly concerning a multifaceted construct like wellbeing, the nuanced comprehension and interpretation of the underlying concept assume paramount importance, which is deeply rooted in the cultural lenses and perspectives one has been exposed to (Klenowski, 2009). Consequently, it is imperative that the viability of the scale in a specific cultural context be substantiated by subjecting it to examination among participants raised within that socio-cultural framework, so as to ensure that the cultural and contextual assumptions intrinsic to that culture are appropriately captured (Keegan et al., 2014).

Historically, the distinction between hedonic and eudaimonic wellbeing has dominated wellbeing research, with the two perspectives often portrayed as competing conceptions of the good life. More recent scholarship, however, increasingly advocates a multidimensional understanding in which hedonic and eudaimonic wellbeing are viewed as complementary components of overall wellbeing rather than mutually exclusive constructs (Delle Fave et al., 2011; Li and Shen, 2026). Consistent with this perspective, several well-established wellbeing measures integrate both traditions within a broader assessment framework. For example, the Warwick–Edinburgh Mental wellbeing Scale (Tennant et al., 2007) assesses both hedonic and eudaimonic facets of wellbeing, while the Scale of General Wellbeing (Longo et al., 2016) adopts a unified framework encompassing multiple domains of positive functioning. The WeBS similarly follows this multidimensional approach by assessing hedonic and eudaimonic wellbeing alongside financial, physical, and social wellbeing within a higher-order model of subjective wellbeing.

Despite the popularity of research on wellbeing in the nation, Singh et al. (2016a,b) observed a notable limitation in these endeavors in India - the lack of rigorous validation of employed measurement scales. The authors further emphasize that the assumption of similar psychometric properties for selected scales in the Indian context cannot be taken for granted (Singh et al., 2016a,b). Although this is the case, a handful of wellbeing scales tailored to the Indian population have been developed (e.g., Nagpal and Sell, 1985; Verma and Verma, 1989; Bhogle and Prakash, 1995) or validated within the Indian milieu (e.g., McIntyre et al., 2019; Mehrotra et al., 2013; Singh et al., 2016a,b, 2018). However, adapting the comprehensive WeBS constructed by Lui and Fernando (2018) to the Indian cultural context, which claims to be “the first self-report measure that utilizes a theory of wellbeing and taps all five important domains of wellbeing and needs satisfaction” (p. 152), could potentially pave the way for elevated standards of quality in wellbeing research investigations and the formulation of wellbeing intervention strategies within the Indian backdrop. Thus, the present study aims to establish the validity of the Wellbeing scale developed by Lui and Fernando (2018) in the Indian context. In addition to that, the study also looks at the different socio-demographic factors and its association with the wellbeing domains presented.

## 2 Materials and methods

### 2.1 Participants

The sample constituted a total of 1795 participants, with the age ranging between 18 to 72 years. Out of 1795, 986 participants responded in English and 809 responded in Hindi, counted as two samples, Sample 1 and 2. Sample 1, constituting English respondents (*N* = 986), included 529 (53.7%) females, 450 (45.6%) males, and 7 (0.7%) participants who preferred not to disclose their gender. Most of the participants were students (72.3%), graduates (62.1%), unmarried (81.4%), resided in urban areas (77.4%), lived in a nuclear family setup (58.2%), identified as Hindu (86.1%), and perceived their family income to be fully sufficient (59.43%). Additionally, Sample 2, comprising Hindi respondents (*N* = 809), comprised 416 (51.4%) females, 383 (47.%) males, and 10 (1.2%) respondents who preferred not to disclose their gender. Similar to Sample 1, a majority of the participants of Sample two were students (57.7%), graduates (46.7%), unmarried (64.2%), resided in urban areas (67.5%), lived in a nuclear family setup (62.5%), identified as Hindu (93.4%), and perceived their family income to be fully sufficient (55.3%).

### 2.2 Measures

#### 2.2.1 Wellbeing Scale

The Wellbeing Scale (Lui and Fernando, 2018) measures SWB as comprising of a broad general factor and five domain-specific factors, namely financial wellbeing (FW), physical wellbeing (PW), social wellbeing (SW), eudaimonic wellbeing (EW), and hedonic wellbeing (HW). The 29-item scale is rated on a 6-point Likert response format ranging from 1 (Strongly disagree) to 6 (Strongly agree), with the overall WeBS score computed as the average score from all 29 items. The original scale yielded a Cronbach's alpha of 0.92 for the general factor, with Cronbach's alpha values ranging from 0.80 to 0.85 for the five subdomains.

#### 2.2.2 Satisfaction with Life Scale

The Satisfaction with Life Scale (SWLS; Diener et al., 1985) comprise five items that assess global life satisfaction as a measure of SWB. The items are rated on seven-point Likert-type responses format ranging from 1 (Completely disagree) to 7 (Completely agree). The original scale reported good internal consistency (Cronbach's alpha = 0.87) and good 2-month test-retest reliability (*r* = 0.82).

#### 2.2.3 Scale of Positive and Negative Experience

The 12-item Scale of Positive and Negative Experience (SPANE, Diener et al., 2009) measures the constitutes 12 items, with six items each measuring estimate the frequency of includes six it measures the frequency of experiencing positive and negative experiences, negative feelings, and a combined balance score. The items are rated on a five-point scale ranging from 1 (Very rarely or never) to 5 (Very often or always). The original scale reported good internal consistency reliability and moderate 1-month test-retest reliability, with Cronbach's alpha 0.84, 0.80, and 0.88 and test-retest reliability indices of 0.62, 0.62, and 0.68 for the positive, negative, and balance scores, respectively.

### 2.3 Instrument Translation

The SWLS and SPANE, although originally developed in English, have previously demonstrated acceptable psychometric properties among Indian samples. The SWLS has extensively been used as a measure of life satisfaction among Indian samples (For example, Agrawal et al., 2011; Banjare et al., 2015; Tiwari et al., 2018; Suar et al., 2019; Varma et al., 2007), while the Hindi version of the SWLS has been validated by Maheshwari and Singh (2009), Mishra and Dixit (2017), Singh et al. (2020), and Singh et al. (2021). Similarly, the SPANE has consistently been used as a measure of positive and negative experience and affect and the Hindi version of the scale has been validated by Mishra and Dixit (2017) and Singh et al. (2016a,b). Two bilingual experts independently translated the items from English to Hindi, and two different bilingual experts back-translated the Hindi items into English. Minor discrepancies were discussed and resolved, after which the Hindi version was finalized and approved by the first author and the bilingual experts.

### 2.4 Data Collection

The participants were recruited using convenience sampling over a period of 5 months starting from May 2022 to September 2022. Multiple recruitment channels were used, such as class WhatsApp groups and brief announcements delivered by course instructors in various colleges and universities. The interested participants were briefed about the purpose of the research and voluntary nature of participation. They were also assured that their responses would remain confidential and that they were free to withdraw their participation at any point. Data was collected using a Google Form, prepared in both English and Hindi. The current study is part of a broader project focusing on happiness and wellbeing, and the project survey's entirety spanned 30 to 40 min. The Google Form comprised a consent form, demographic information sheet, and the selected measures. The interested and consenting participants could choose to respond in either of the languages depending on their preference. All questions in the survey were mandatory; therefore, there were no missing data or partial responses.

The study was reviewed and approved by the Institute Ethics Committee of the authors' institutional affiliation. Written informed consent was obtained from all participants, clearly stating confidentiality, anonymity, and the voluntary nature of participation. No identifying information was collected at any stage of the research.

### 2.5 Statistical Analysis

To evaluate the psychometric properties of the WeBS, confirmatory factor analysis (CFA) was conducted to compare four models constituting a unidimensional structure, five factor structure, higher order factor structure, and bifactor structure. Following which, the descriptive statistics, reliability analysis, and convergent and divergent validity were estimated based on the procedure suggested by Fornell and Larcker (1981). To estimate the convergent and discriminant validity of the WeBS, correlations were also computed with scores on SWLS and SPANE. Measurement invariance was conducted across language and five demographic variables, including age, gender, education, income sufficiency, and area of residence. Additionally, multivariate analysis of variance (MANOVA) was conducted to examine the role of socio-demographic variables. The benchmarks for interpreting the effect size (partial eta squared) were 0.01, 0.06, and 0.14, which represented a small, medium, and large effect size, respectively (Cohen, 2013). SPSS was used to estimate the descriptive statistics and MANOVA, while the SemTool, SemPlot, and Lavaan packages in R (Version 4.3.2) were used to conduct CFA and measurement invariance testing.

## 3 Results

### 3.1 Factor Structure of the WeBS

At the outset, preliminary item analyses were conducted for the English version (*n* = 986) and the translated Hindi version (*n* = 809) of the WeBS. The item means and SD ranged from 3.79 to 4.56 and 1.41 to 1.64 for Sample 1 and 4 to 4.45 and 1.24 to 1.39 for Sample 2. Additionally, to check for non-normal distribution of scores, the absolute skewness and kurtosis were examined. The skewness for Sample 1 and 2 ranged from −0.77 to −0.22 and −0.83 to −0.43, respectively, while the kurtosis ranged from −1.14 to −0.45 for Sample 1 and −0.60 to 0.22 for Sample 2. Given that the skewness (< 2) and kurtosis (< 7) were within the acceptable range (Byrne, 2010; Hair et al., 2019), the results indicate that the scores on the WeBS were approximately normally distributed.

Consistent with the analytical procedure followed by Lui and Fernando (2018), this study examined the model fit indices for a five factor model (M1), single factor model (M2), higher-order factor model (M3), and bifactor model (M4). The benchmarks for the model fit indices were based on the criteria highlighted in the existing literature. For a good model fit, Hu and Bentler (1999) recommend the RMSEA, SRMR, CFI, and TLI values to be < 0.08, < 0.08, ≥0.95, and ≥0.95, respectively. Since the data was approximately normally distributed, robust Maximum Likelihood estimation was used. Table 1 displays the model fit indices for all four factor structures for both Sample 1 and 2.

**Table 1:** Summary of fit indices across four models for WeBS.

Model	χ^2^	*df*	Robust CFI	Robust TLI	Robust RMSEA (90% CI)	SRMR	AIC	BIC
*Sample 1*								
M1. Five-factor	2227.518	367	0.925	0.917	0.068 (0.064–0.071)	0.05	83821.961	84154.73
M2. Single factor	3892.942	350	0.840	0.828	0.098 (0.095–0.102)	0.061	82986.088	83260.132
M3. Higher-order factor	2293.451	372	0.922	0.915	0.068 (0.065–0.072)	0.051	83877.894	84186.194
M4. Bifactor	1410.608	353	0.961	0.955	0.05 (0.046–0.054)	0.034	83033.051	83434.331
*Sample 2*								
M1. Five-factor	1306.854	367	0.958	0.954	0.051 (0.046–0.055)	0.034	62086.796	62406.11
M2. Single factor	1688.383	350	0.933	0.928	0.064 (0.06–0.068)	0.038	60539.751	60802.716
M3. Higher-order factor	1388.478	372	0.954	0.95	0.053 (0.048–0.057)	0.035	62158.42	62454.255
M4. Bifactor	970.667	353	0.975	0.972	0.04 (0.035–0.044)	0.027	61778.609	62163.665

*N* = 986 in Sample 1 and *N* = 809 in Sample 2. CFI: comparative fit index; RMSEA, root mean squared error of approximation; SRMR, standardized root mean squared residual; TLI, Tucker–Lewis fit index; AIC, Akaike Information Criterion; BIC, Bayes Information Criterion.

As per the results, the five factor model did not adequately fit the data for Sample 1, with the CFI and TLI values less than 0.95, although the fit indices for Sample 2 indicated a moderate fit. The single factor model did not adequately fit the data either, since the CFI and TLI values for both Sample 1 and 2 were less than 0.95 and the RMSEA for Sample 1 was greater than 0.08. In addition, while the higher-order factor model yielded a good fit for the Sample 2, the CFI and TLI for Sample 1 were marginally below the recommended cut-off of 0.95. The fit indices for the bifactor model were within the recommended range for both Samples 1 and Sample 2. As compared to the higher-order factor model, the bifactor model also depicted smaller AIC and BIC values; thus, indicating a preference for the same. Although these results are consistent with Lui and Fernando's (2018) findings, existing literature focusing on the statistical comparison of bifactor and higher-order factor models suggest that model fit indices tend to be biased in favor of a bifactor structure (Murray and Johnson, 2013; Morgan et al., 2015). Therefore, the subsequent results examine the measurement quality of the bifactor and higher-order factor solutions, assessed in terms of the standardized factors loadings, residual error variances, and factorial intercorrelations between the subscales. The criteria for assessment were based on the recommendations of Morin et al. (2020), who suggest that the factor solutions with significant loadings >0.35, residual error variances ranging between 0.10 to 0.90, and lower factorial intercorrelations between the subscales should be considered favorable.

Tables 2, 3 exhibit the factor loadings and residual error variances for the bifactor and higher-order factor models, respectively. For the bifactor model, the standardized factor loadings ranged from 0.073 (Item 23) to 0.630 (Item 7) and −0.001(Item 18) to 0.782 (Item 13) for the subdomains for Samples 1 and 2, respectively. The factor loadings for 10 items for Sample 1 and 16 items for Sample 2 were less than 0.35. Further, the factor loadings for the general factor ranged from 0.513 (Item 7) to 0.823 (Item 14) and 0.595 (Item 3) to 0.812 (Item 22) for Samples 1 and 2, respectively. Furthermore, the item uniqueness, measured in terms of the residual error variances, ranged from 0.206 (Item 29) to 0.514 (Item 24) for Sample 1 and −0.151 (Item 13) to 0.456 (Item 24) for Sample 2. In contrast, the standardized factor loadings for the higher-order factor model ranged from 0.704 (Item 24) to 0.851 (Item 28) for Sample 1 and 0.678 (Item 3) to 0.841 (Item 27). The factor loadings of the first-order factors on the second-order factor ranged from 0.801 (PW) to 0.969 (EW) and 0.944 (FW) to 0.979 (SW) for Samples 1 and 2, respectively. Measures of item uniqueness for the higher-order factor model were also within the recommended criteria, with residual error variances for Sample 1 ranging from 0.276 (Item 28) to 0.504 (Item 24) and for Sample 2 ranging from 0.293 (Item 27) to 0.54 (Item 3). Therefore, the higher-order structure of the WeBS reported better measurement quality, as compared to the bifactor structure.

**Table 2:** Factor loadings and residual error variances for the bifactor model.

Item	Sample 1							Sample 2						
	FW	PW	SW	EW	HW	SWB	δ	FW	PW	SW	EW	HW	SWB	δ
WeBS1		0.471				0.607	0.410		0.320				0.700	0.408
WeBS2	0.606					0.630	0.236	0.538					0.629	0.315
WeBS3	0.604					0.608	0.265	0.518					0.595	0.378
WeBS4	0.371					0.676	0.406	0.294					0.687	0.441
WeBS5	0.377					0.612	0.483	0.391					0.657	0.416
WeBS6		0.500				0.594	0.397		0.319				0.713	0.389
WeBS7		0.630				0.513	0.340		0.457				0.623	0.404
WeBS8		0.389				0.715	0.338		0.155				0.775	0.376
WeBS9		0.571				0.606	0.307		0.243				0.761	0.362
WeBS10						0.747	0.441						0.798	0.363
WeBS11			0.465			0.657	0.351			0.137			0.75	0.745
WeBS12			0.369			0.761	0.284			0.062			0.79	0.790
WeBS13			0.391			0.724	0.323			0.782			0.73	0.734
WeBS14						0.823	0.323						0.768	0.41
WeBS15						0.717	0.485						0.795	0.369
WeBS16		0.374				0.702	0.368		0.214				0.77	0.769
WeBS17						0.779	0.393						0.778	0.394
WeBS18			0.116			0.756	0.415			−0.001			0.803	0.354
WeBS19						0.819	0.330						0.790	0.376
WeBS20				0.319		0.787	0.279				0.359		0.79	0.789
WeBS21				0.379		0.775	0.256				0.161		0.76	0.755
WeBS22				0.265		0.780	0.321				0.284		0.81	0.812
WeBS23				0.073		0.697	0.508				0.147		0.79	0.785
WeBS24				0.135		0.684	0.514				0.038		0.74	0.737
WeBS25				0.093		0.760	0.414				0.029		0.79	0.794
WeBS26				0.114		0.734	0.449						0.75	0.748
WeBS27					0.117	0.821	0.313					0.222	0.80	0.802
WeBS28					0.313	0.799	0.264					0.246	0.77	0.772
WeBS29					0.116	0.781	0.206					0.256	0.76	0.763

**Table 3:** Factor loadings and residual error variances for the higher-order factor model.

Item	Sample 1						Sample 2					
	FW	PW	SW	EW	HW	δ	FW	PW	SW	EW	HW	δ
WeBS1		0.774				0.401		0.761				0.421
WeBS2	0.767					0.412	0.705					0.503
WeBS3	0.758					0.426	0.678					0.54
WeBS4	0.783					0.387	0.748					0.441
WeBS5	0.712					0.493	0.723					0.477
WeBS6		0.775				0.399		0.771				0.405
WeBS7		0.762				0.419		0.704				0.504
WeBS8		0.821				0.325		0.799				0.361
WeBS9		0.812				0.341		0.802				0.356
WeBS10				0.758		0.426				0.796		0.366
WeBS11			0.757			0.427			0.765			0.415
WeBS12			0.849			0.278			0.807			0.348
WeBS13			0.810			0.345			0.760			0.422
WeBS14				0.817		0.333				0.778		0.395
WeBS15	0.740					0.452	0.806					0.350
WeBS16		0.794				0.370		0.802				0.357
WeBS17				0.773		0.402				0.776		0.398
WeBS18			0.770			0.407			0.809			0.345
WeBS19	0.761					0.421	0.790					0.376
WeBS20				0.818		0.331				0.817		0.333
WeBS21				0.813		0.339				0.768		0.411
WeBS22				0.806		0.350				0.835		0.303
WeBS23				0.706		0.502				0.801		0.358
WeBS24				0.704		0.504				0.742		0.450
WeBS25				0.772		0.404				0.797		0.364
WeBS26				0.752		0.435				0.761		0.421
WeBS27					0.842	0.291					0.841	0.293
WeBS28					0.851	0.276					0.815	0.337
WeBS29					0.848	0.281					0.817	0.332
SWB	0.905	0.801	0.908	0.969	0.947		0.944	0.945	0.979	0.977	0.950	

### 3.2 Reliability

The WeBS reported Cronbach's alpha ranging from 0.79 to 0.85 for the five domain specific factors and 0.92 for the overall WeBS score in the initial validation conducted by Lui and Fernando (2018). The Cronbach's alpha of the overall WeBS score in this study was 0.97 for both English and Hindi respondents, and the Cronbach's alpha of the five domains ranged from 0.87 to 0.91 and 0.86 to 0.92 for the English and Hindi respondents, respectively. Although Cronbach's alpha is commonly reported as a measure of internal consistency, Cronbach's alpha may overestimate or underestimate the reliability of multidimensional constructs (Al Nima et al., 2020). To overcome the limitations of Cronbach's alpha, Omega hierarchical and Omega higher-order are recommended as reliable estimates of internal consistency for bifactor and higher-order factor models, respectively (Flora, 2020; Reise et al., 2023). Therefore, Omega hierarchical and Omega higher-order were estimated along with Cronbach's alpha.

Table 4 displays the reliability estimates for the bifactor and higher-order factor models for both samples. For the bifactor model, the omega hierarchical was 0.93 for Sample 1 and 0.96 for Sample 2, which indicates that the general factor accounts for 93% and 96% of the total variance over and above the influence of the specific factors. The omega hierarchical subscale scores represent the proportion of variance in the subscale that corresponds to the specific factor, over and above the influence of the general factor. The omega hierarchical subscale ranged from 0.01 to 0.33 for Sample 1 and 0.01 to 0.27 for Sample 2, which suggests that the subdomains correlate more strongly with the general factor SWB instead of the intended specific factors as subdimensions of SWB. For the higher-order factor model, the omega higher-order was 0.93 for Sample 1 and 0.96 for Sample 2, which indicates that an over-arching construct of SWB accounts for 93% and 96% of the scale's total score variance. The omega coefficient for five first-order factors ranged from 0.87 to 0.94 and 0.86 to 0.94 for Samples 1 and 2, respectively. Thus, the results indicate that the variance of the subscale scores is explained by the five first-order factors.

**Table 4:** Reliability estimates for the bifactor and higher-order factor models.

Factors	Bifactor		Higher-order	
	Sample 1	Sample 2	Sample 1	Sample 2
	ω_H − ss/_ω_H_	ω_H − ss/_ω_H_	ω_u_/ω_Ho_	ω_u_/ω_Ho_
FW	0.33	0.27	0.88	0.88
PW	0.35	0.12	0.91	0.90
SW	0.16	0.10	0.87	0.86
EW	0.06	0.04	0.94	0.94
HW	0.01	0.01	0.88	0.86
SWB	0.93	0.96	0.93	0.96

ω_H_, Omega hierarchical; ω_H − ss_, Omega hierarchical subscales; ω_Ho_, Omega higher-order; ω, ω_u:_ Omega unidimensional.

### 3.3 Validity

The convergent and divergent validity of the bifactor and higher-order factor models were estimated based on the procedure suggested by Fornell and Larcker (1981). Convergent validity was estimated by examining the average variance extracted (AVE) and composite reliability (CR), and divergent validity was estimated by comparing the square root of AVE and the inter-correlations among the five first order factors and the second order factor. To ensure convergent validity, the AVE should be greater than or equal to 0.50, lower than the CR, and the CR should be greater than 0.70 (Fornell and Larcker, 1981; Hair et al., 2019). In addition, the Fornell and Larcker (1981) criterion for divergent validity requires the square root of AVE to be greater than the correlation among the latent variables. Tables 5, 6 display the values of CR, AVE, square root of AVE, and inter-factor correlations for the bifactor and higher-order factor models, respectively.

**Table 5:** Inter-correlations, CR, AVE, and square root of AVE for the bifactor model.

Sl. No.	Factors	CR	AVE	1	2	3	4	5	6
*Sample 1 (n = 986)*									
1	FW	0.562	0.253	**0.503**					
2	PW	0.656	0.248	0.575^**^	**0.498**				
3	SW	0.341	0.130	0.573^**^	0.620^**^	**0.360**			
4	EW	0.222	0.052	0.608^**^	0.677^**^	0.767^**^	**0.227**		
5	HW	0.206	0.094	0.661^**^	0.663^**^	0.739^**^	0.837^**^	**0.306**	
6	SWB	0.968	0.515	0.700^**^	0.842^**^	0.865^**^	0.920^**^	0.883^**^	**0.717**
*Sample 2 (n = 809)*									
1	FW	0.486	0.199	**0.446**					
2	PW	0.348	0.090	0.753^**^	**0.301**				
3	SW	0.222	0.159	0.711^**^	0.807^**^	**0.398**			
4	EW	0.156	0.038	0.685^**^	0.814^**^	0.837^**^	**0.196**		
5	HW	0.185	0.072	0.669^**^	0.756^**^	0.794^**^	0.860^**^	**0.269**	
6	SWB	0.974	0.562	0.825^**^	0.921^**^	0.917^**^	0.941^**^	0.888^**^	**0.750**

CR, Composite reliability; AVE, Average Variance Extracted; Square roots of AVE is mentioned in bold. ^*^Correlation is significant at the 0.05 level (2-tailed) and ^**^Correlation is significant at the 0.01 level (2-tailed).

**Table 6:** Inter-correlations, CR, AVE, and square root of AVE for the higher-order factor model.

Sl. No.	Factors	CR	AVE	1	2	3	4	5	6
*Sample 1 (n = 986)*									
1	FW	0.888	0.568	**0.754**					
2	PW	0.905	0.614	0.699^**^	**0.783**				
3	SW	0.874	0.636	0.746^**^	0.620^**^	**0.797**			
4	EW	0.937	0.597	0.780^**^	0.713^**^	0.803^**^	**0.773**		
5	HW	0.884	0.717	0.755^**^	0.663^**^	0.739^**^	0.848^**^	**0.847**	
6	SWB	0.959	0.824	0.894^**^	0.842^**^	0.865^**^	0.948^**^	0.883^**^	**0.908**
*Sample 2 (n = 809)*									
1	FW	0.881	0.552	**0.743**					
2	PW	0.899	0.599	0.818^**^	**0.774**				
3	SW	0.866	0.617	0.802^**^	0.807^**^	**0.786**			
4	EW	0.942	0.620	0.806^**^	0.842^**^	0.861^**^	**0.788**		
5	HW	0.864	0.680	0.762^**^	0.756^**^	0.794^**^	0.859^**^	**0.824**	
6	SWB	0.983	0.920	0.909^**^	0.921^**^	0.917^**^	0.961^**^	0.888^**^	**0.959**

CR, Composite reliability; AVE, Average Variance Extracted; Square roots of AVE is mentioned in bold. ^*^Correlation is significant at the 0.05 level (2-tailed) and ^**^Correlation is significant at the 0.01 level (2-tailed).

As per the results for the bifactor model, the CR estimates for both Samples 1 and 2 were less than 0.70 and the AVE values were less than 0.50, except for the general factor. Additionally, the square root of AVE for both samples was less than the inter-factor correlations. Therefore, the results indicate that the bifactor model does not satisfy the criteria for convergent and discriminant validity. In contrast, the results for the higher-order factor model for both samples indicate that the CR estimates were greater than 0.70 and the AVE values were greater than 0.50; therefore, the results are indicative of convergent validity. However, the square root of AVE was not greater than the inter-factor correlations, which indicates that the higher-order factor structure does not meet the criterion for discriminant validity.

Based on the factor loadings and estimates of reliability and validity, the higher-order factor model seems to be more favorable, as compared to the bifactor model. Therefore, subsequent analysis, including construct validity assessment and measurement invariance testing, will be based on the higher-order factor structure of the WeBS.

Correlation analysis was conducted to evaluate the construct validity of the WeBS. It was hypothesized that the overall WeBS score and the first-order factors would be positively correlated with life satisfaction and positive experience and negatively correlated with negative experience. Table 7 displays the correlations among overall WeBS and the subdomains with the validating scales for both Samples 1 and 2.

**Table 7:** Correlations among WeBS and validating scales.

Sl. No.	Scales/ Sub-scales	1	2	3	4	5	6	7	8	9	10
1	SWB		0.909^**^	0.921^**^	0.917^**^	0.961^**^	0.888^**^	0.748^**^	0.631^**^	−0.015	0.399^**^
2	FW	0.894^**^		0.818^**^	0.802^**^	0.806^**^	0.762^**^	0.738^**^	0.591^**^	0.056	0.322^**^
3	PW	0.842^**^	0.699^**^		0.807^**^	0.842^**^	0.756^**^	0.698^**^	0.576^**^	0.008	0.348^**^
4	SW	0.865^**^	0.746^**^	0.620^**^		0.861^**^	0.794^**^	0.665^**^	0.579^**^	−0.037	0.383^**^
5	EW	0.948^**^	0.780^**^	0.713^**^	0.803^**^		0.859^**^	0.687^**^	0.592^**^	−0.038	0.391^**^
6	HW	0.883^**^	0.755^**^	0.663^**^	0.739^**^	0.848^**^		0.662^**^	0.583^**^	−0.080^*^	0.417^**^
7	SWLS	0.719^**^	0.704^**^	0.593^**^	0.589^**^	0.652^**^	0.675^**^		0.591^**^	0.019	0.349^**^
8	SPANE-P	0.690^**^	0.610^**^	0.588^**^	0.582^**^	0.638^**^	0.670^**^	0.697^**^		−0.099^**^	0.687^**^
9	SPANE-N	−0.061	−0.008	−0.075^*^	−0.048	−0.039	−0.155^**^	−0.023	−0.097^**^		−0.791^**^
10	SPANE-B	0.506^**^	0.416^**^	0.447^**^	0.425^**^	0.456^**^	0.557^**^	0.485^**^	0.739^**^	−0.742^**^	

Correlation coefficients for Sample 1 (*n* = 986) are listed from left to right. Correlation coefficients for Sample 2 (*n* = 809) are listed from right to left. SWB, Higher-order factor; FW, financial wellbeing; PW, physical wellbeing; SW, social wellbeing; EW, eudaimonic wellbeing; HW, hedonic wellbeing; SWLS, satisfaction with life scale; SPANE-P, positive feelings; SPANE-N, negative feelings; SPANE-B, overall affect balance score. ^*^Correlation is significant at the 0.05 level (2-tailed) and ^**^Correlation is significant at the 0.01 level (2-tailed).

As per the results, overall WeBS and the five subdomains reported significant positive correlations with life satisfaction and positive experience for both samples. However, only PW and HW reported significant negative correlations with negative experience for Sample 1, while only HW reported a significant negative correlation with negative experience for Sample 2. Although the correlation between PW and negative experience reached statistical significance, the strength of the association was negligible (*r* = −0.075, *p* < 0.05), and hence, not substantive in practical terms. Therefore, the hypothesis, which stated overall WeBS and the subdomains would be positively correlated with life satisfaction and positive experience and negatively correlated with negative experience was only partially approved.

### 3.4 Measurement Invariance

Measurement invariance testing was conducted across language and across five demographic variables, including age, gender, education, income sufficiency, and area of residence. Table 8 displays the results of the measurement invariance analysis. Existing literature suggests ΔCFI values < 0.01 and ΔRMSEA ≤ 0.015 as indicators of measurement invariance (Chen, 2007; Cheung and Rensvold, 2002; Hirschfeld and Von Brachel, 2019). Four steps of measurement invariance testing were conducted, which include configural invariance that assesses equivalence of model form; metric invariance that examines equivalence across factor loadings; scalar invariance focusing on equivalence of item intercepts; and lastly, residual invariance that tests the equivalence of items residuals (Putnick and Bornstein, 2016). The series of model comparisons indicate that the specified models for language and income achieved scalar, while the specified models for age, education, gender, and residence achieved residual. Therefore, the results provide strong support for the measurement invariance of the higher-order factor structure of the WeBS across language and demographic variables.

**Table 8:** Measurement invariance testing across language and socio-demographic variables.

Variable	χ^2^	df	*p*	*Δχ* ^2^	Δdf	Robust RMSEA	ΔRMSEA	Robust CFI	ΔCFI
*Language*									
Configural	3681.929	744	< 0.01			0.062		0.937	
Metric	3738.93	772	< 0.01	57.001	28	0.061	−0.001	0.936	−0.001
Scalar	3922.139	795	< 0.01	183.209	23	0.062	0.001	0.932	−0.004
Residual	4335.301	824	< 0.01	413.162	29	0.065	0.003	0.923	−0.009
*Age*									
Configural	4332.275	1116	< 0.01			0.064		0.931	
Metric	4390.111	1172	< 0.01	57.836	56	0.063	−0.001	0.931	0
Scalar	4513.829	1218	< 0.01	123.718	46	0.062	−0.001	0.929	−0.002
Residual	4689.766	1276	< 0.01	175.937	58	0.062	0	0.927	−0.002
*Education*									
Configural	4271.245	1116	< 0.01			0.064		0.932	
Metric	4341.898	1172	< 0.01	70.653	56	0.063	−0.001	0.931	−0.001
Scalar	4401.1	1218	< 0.01	59.202	46	0.062	−0.001	0.931	0
Residual	4563.868	1276	< 0.01	162.768	58	0.061	−0.001	0.929	−0.002
*Gender*									
Configural	3801.241	744	< 0.01			0.063		0.933	
Metric	3842.304	772	< 0.01	41.063	28	0.062	−0.001	0.933	0
Scalar	3893.226	795	< 0.01	50.922	23	0.062	0	0.932	−0.001
Residual	3954.427	824	< 0.01	61.201	29	0.061	−0.001	0.932	0
*Residence*									
Configural	3760.388	744	< 0.01			0.062		0.935	
Metric	3801.911	772	< 0.01	41.523	28	0.061	−0.001	0.934	−0.001
Scalar	3862.855	795	< 0.01	60.944	23	0.061	0	0.933	−0.001
Residual	3951.575	824	< 0.01	88.72	29	0.06	−0.001	0.932	−0.001
*Income*									
Configural	4321.404	1116	< 0.01			0.063		0.932	
Metric	4370.623	1172	< 0.01	49.219	56	0.062	−0.001	0.932	0
Scalar	4535.183	1218	< 0.01	164.56	46	0.062	0	0.929	−0.003
Residual	4844.525	1276	< 0.01	309.342	58	0.063	0.001	0.924	−0.005

### 3.5 Role of Socio-demographic variables

Multivariate analysis was conducted to examine whether the scores on the WeBS varied across socio-demographic variables, including age, gender, educational qualification, occupation, marital status, religious affiliation, residence type, income sufficiency, and family type. Since the results of measurement invariance indicate strong equivalence across language; therefore, the data from the two samples was combined for the multivariate analysis.

The results of the multivariate analysis revealed a significant main effect for education (Pillai's trace = 0.017, F(15, 5295) = 1.974, *p* < 0.05, partial η2 = 0.01), family income (Pillai's trace = 0.077, F(10, 3528) = 14.117, *p* < 0.01, partial η2 = 0.04), and residence type (Pillai's trace = 0.011, F(5, 1763) = 3.968, *p* < 0.01, partial η2 = 0.01), with effect sizes ranging from 0.01 to 0.04 (see Table 9). Further, *post-hoc* tests were conducted to examine the differences between the groups in demographic variables of significance. With regards to education status, graduate (*n* = 990) and post-graduate (*n* = 449) participants reported significantly higher scores on the second order factor and all five subdomains as compared to participants with high school level education or below (*n* = 260). As for the family income, participants who perceived the family income to be fully sufficient (*n* = 1033) scored significantly higher on the overall WeBS score and five subscales in comparison to participants who perceived the family income to be partially sufficient (*n* = 557) or not sufficient (*n* = 205). Additionally, participants who perceived the family income to be partially sufficient scored significantly higher on financial and hedonic wellbeing, as compared to participants who perceived the family income to be not sufficient. Lastly, with regards to the residence type, participants residing in urban areas (*n* = 1309) scored significantly higher on financial wellbeing, as compared to participants residing in rural areas (*n* = 486). The mean, SD, and results of univariate effects for education, income sufficiency, and residence type are summarized in Supplementary Tables 1, 2.

**Table 9:** Main Effect of Demographic Variables.

Effect	Pillai's Trace	*F*	*Df*	Error df	Sig	Partial eta squared
Age	0.006	1.019	10	3528	0.425	0.003
Gender	0.009	1.524	10	3528	0.124	0.004
Education	0.017	1.974	15	5295	0.014	0.006
Occupation	0.008	0.962	15	5295	0.493	0.003
Marital status	0.008	0.719	20	7064	0.810	0.002
Religion	0.012	0.729	30	8835	0.858	0.002
Residence type	0.011	3.968	5	1763	0.001	0.011
Income level	0.077	14.117	10	3528	0.000	0.038
Family type	0.015	1.368	20	7064	0.126	0.004

## 4 Discussion

This study presents the psychometric properties of the English and Hindi translated version of the WeBS in the Indian context. Consistent with the findings of the original study by Lui and Fernando (2018), the results confirmed that the bifactor model reported the best fit for the original English version and the Hindi translated version of the WeBS.

To the best of our knowledge, this is the first study to examine the psychometric properties and factor structure of the WeBS in the Indian milieu. Since this study also aimed to validate the Hindi translated version of the scale, measurement invariance analysis was conducted, which revealed strong invariance across language. Additionally, the bifactor structure of the WeBS demonstrated good reliability and validity, with the overall WeBS score and the five subdomains reporting significant positive correlations with life satisfaction and positive experience. The overall WeBS score reported strong positive associations with life satisfaction and positive experience, which is consistent with Diener's (1984) tripartite conceptualization of SWB, comprising positive evaluations of one's life, positive affect, and negative affect. Further, the results indicate strong positive associations between FW and life satisfaction, which is also consistent with past findings. For instance, Dickason-Koekemoer and Ferreira (2019) reported a strong positive association between investor's perceived financial wellbeing and life satisfaction. Yeo and Lee (2019) in their study reported a significant direct effect of financial wellbeing on life satisfaction. However, only PW and HW for Sample 1 and only HW for Sample 2 reported significant negative correlations with negative experience. In contrast, past findings have reported negative associations between measures of wellbeing and negative experience (e.g, Du Plessis and Guse, 2017; Jovanović, 2015a; Kyriazos et al., 2018; Rahm et al., 2017; Singh et al., 2018).

This study also explored whether the scores on the WeBS and its subdomains varied across socio-demographic variables. The results of multivariate analysis revealed significant main effects for education, family income, and place of residence. The univariate analysis indicated that graduate and undergraduate participants scored significantly higher as compared to participants with high school level education or below, which is consistent with past findings that suggest a strong link between education status and wellbeing. For instance, Jongbloed (2018) found tertiary education to be a significant predictor of psychological, emotional, and mental wellbeing. Similarly, Nikolaev (2018) reported higher levels of eudaimonic and hedonic wellbeing among people with higher education. Education is commonly associated with higher levels of wellbeing, since education may enhance wellbeing because of factors such as higher income, employment opportunities, and job security (Helliwell et al., 2012). With regards to family income, the results revealed higher scores for participants who perceived the family income to be fully sufficient, which is in line with past research suggesting a strong connection between income and measures of wellbeing. Gildner et al. (2019) reported a positive relationship between perceived income adequacy and quality of life. Likewise, Pereira and Coelho (2013) in their study found strong effects of perceived income adequacy on SWB, stemming from a sense of satisfaction from being able to fulfill basic needs. Recently, Muhammad et al. (2021) found that individuals with partially sufficient income were more likely to suffer from low SWB as opposed to those with income that is sufficient to meet their needs. Lastly, the univariate results suggest participants residing in urban areas scored higher in comparison to those residing in rural areas. However, these findings could be attributed to the majority of the participants from both samples being residents of urban areas. Nevertheless, recent research focusing on the effect of urban living conditions on measures of SWB suggests that factors such as access to nature, quality of housing, and quality of public goods exhibit strong associations with SWB (Rüger et al., 2023).

### 4.1 Limitations and future directions

Although the findings of this research make significant contributions by validating the English and Hindi version of the WeBS in the Indian context, it is essential to consider these findings within the context of their limitations. The participants were recruited using convenience sampling technique and the sample predominantly comprised participants who were students, unmarried, identified as Hindu, and resided in urban areas; hence, the extent to which the findings can be generalized may be limited. Since the sample may not be representative of the general population, there is a need to further investigate whether scores on the WeBS differ across other socio-demographic variables. Additionally, the reliability analyses revealed omega estimates of 0.93 and 0.96 for the hiher-order factor, with the corresponding omega coefficients for the five first-order factors ranging between 0.86 and 0.94. While these estimates satisfy the recommended criterion in existing literature, which suggests that internal consistency estimates greater than 0.95 may be indicative of items that are redundant (Streiner, 2003). However, future attempts to examine the psychometric properties of the WeBS should specifically focus on checking for redundant items and propose a shorter version of the scale. Nevertheless, validating the Hindi translated version of the scale is a significant contribution of this research.

## Data Availability

The raw data supporting the conclusions of this article will be made available by the authors, without undue reservation.
