# Supplementary material for: A new lens on wellbeing: psychometric evaluation of the Wellbeing scale in India
Source: Front Psychol. 2026 Oct 7;17:1898104. doi: 10.3389/fpsyg.2026.1898104 (PMC13682999; doi:10.3389/fpsyg.2026.1898104)
Supplement: Supplementary file 1 [file Table_1.docx]

**Supplementary Table 1**

*Descriptive statistics across demographic variables*

| Variable | | N | FW | | PW | | SW | | EW | | HW | |
| --- | --- | --- | --- | --- | --- | --- | --- | --- | --- | --- | --- | --- |
|  |  |  | x̄ | SD | x̄ | SD | x̄ | SD | x̄ | SD | x̄ | SD |
| **Education** | |  |  |  |  |  |  |  |  |  |  |  |
|  | High school and below | 260 | 3.88 | 1.24 | 3.83 | 1.26 | 4.07 | 1.35 | 3.97 | 1.16 | 3.95 | 1.27 |
|  | Graduation | 990 | 4.23 | 1.15 | 4.14 | 1.21 | 4.43 | 1.23 | 4.37 | 1.09 | 4.34 | 1.25 |
|  | Post-graduation or higher | 449 | 4.26 | 1.12 | 4.16 | 1.19 | 4.42 | 1.2 | 4.37 | 1.12 | 4.38 | 1.24 |
|  | Other | 96 | 4.28 | 0.924 | 4.3 | 0.922 | 4.3 | 1.02 | 4.37 | 0.918 | 4.36 | 1.04 |
| **Residence type** | |  |  |  |  |  |  |  |  |  |  |  |
|  | Rural | 486 | 3.92 | 1.22 | 4.05 | 1.3 | 4.19 | 1.37 | 4.16 | 1.24 | 4.14 | 1.36 |
|  | Urban | 1309 | 4.29 | 1.1 | 4.13 | 1.17 | 4.43 | 1.17 | 4.37 | 1.05 | 4.35 | 1.19 |
| **Income level** | |  |  |  |  |  |  |  |  |  |  |  |
|  | Not sufficient | 205 | 3.59 | 1.27 | 3.88 | 1.3 | 4.04 | 1.4 | 4.04 | 1.26 | 3.89 | 1.39 |
|  | Partially sufficient | 557 | 3.94 | 1.03 | 3.93 | 1.11 | 4.23 | 1.21 | 4.19 | 1.07 | 4.16 | 1.18 |
|  | Fully sufficient | 1033 | 4.44 | 1.11 | 4.25 | 1.22 | 4.5 | 1.19 | 4.44 | 1.08 | 4.45 | 1.22 |

**Supplementary Table 2**

*Effect of Demographic Variables on overall SWB and the five subdomains*

| **Variables:** | **FW** | **PW** | **SW** | **EW** | **HW** |
| --- | --- | --- | --- | --- | --- |
| **Education** |  |  |  |  |  |
| *F* | 4.323 | 4.332 | 4.853 | 7.708 | 5.799 |
| Df | 3 | 3 | 3 | 3 | 3 |
| *P* | 0.005 | 0.005 | 0.002 | 0 | 0.001 |
| Partial Eta Squared | 0.007 | 0.007 | 0.008 | 0.013 | 0.01 |
| **Residence type** |  |  |  |  |  |
| *F* | 4.174 | 0.525 | 2.153 | 2.46 | 0.341 |
| Df | 1 | 1 | 1 | 1 | 1 |
| *P* | 0.041 | 0.469 | 0.142 | 0.117 | 0.559 |
| Partial Eta Squared | 0.002 | 0 | 0.001 | 0.001 | 0 |
| **Income level** |  |  |  |  |  |
| *F* | 50.307 | 14.671 | 10.83 | 9.584 | 14.785 |
| Df | 2 | 2 | 2 | 2 | 2 |
| *P* | 0 | 0 | 0 | 0 | 0 |
| Partial Eta Squared | 0.054 | 0.016 | 0.012 | 0.011 | 0.016 |
